# Verbal memory dysfunction in substance use and gambling addictive disorders: a comparative analysis of performance accuracy and error typologies

**DOI:** 10.3389/fpsyg.2025.1677401

**Published:** 2025-11-13

**Authors:** Davide Crivelli, Doriana Losasso, Simona Riccardi, Maria Francesca Scaramuzzino, Gianmaria Zita, Michela Balconi

**Affiliations:** 1 International research center for Cognitive Applied Neuroscience (IrcCAN), Faculty of Psychology, Università Cattolica del Sacro Cuore, Milan, Italy; 2 Department of Psychology, Università Cattolica del Sacro Cuore, Milan, Italy; 3 Centre of Addiction Treatment, Department of Mental Health and Addiction, ASST Fatebenefratelli-Sacco, Milan, Italy

**Keywords:** substance use disorder, gambling disorder, addiction, neurocognitive screening, memory, learning, error analysis

## Abstract

Addiction is increasingly recognized as a disorder involving not only reward dysregulation but also alterations in core cognitive processes, including learning and memory. Although various studies have documented memory impairments in substance use and behavioral addictions, evidence remains inconsistent—particularly regarding verbal memory deficits and the diagnostic relevance of specific recall error types. This study investigated immediate and delayed verbal memory performance and error patterns in 515 individuals, including patients with stimulant use disorder, alcohol use disorder, cannabis use disorder, or gambling disorder, and a healthy control group. Participants completed a digitized neurocognitive screening battery, which provided not only global performance indices but also specific error metrics. ANCOVA models controlling for age and sex revealed significantly lower correct recall scores in stimulant, alcohol, and gambling disorder groups compared to controls, across immediate and delayed trials. Alcohol use disorder was associated with elevated intrusion errors—suggesting inhibitory and interference suppression deficits—while stimulant use was linked to increased repetition errors—suggesting impaired monitoring and impulsive retrieval. Gambling disorder mirrored the overall performance decline of substance-related disorders but did not exhibit elevated error rates, pointing to a partially distinct cognitive profile. These findings highlight the value of combining global performance measures with qualitative error analysis in the assessment of memory function in addiction. By differentiating between types of recall errors, clinicians may better identify disorder-specific cognitive signatures, supporting more refined diagnostics and intervention strategies. The results further support the applicability of advanced neurocognitive tools for neuropsychological assessment in psychiatry.

## Introduction

Addiction, whether driven by chronic substance use or compulsive behaviors, is increasingly conceptualized not only as a disorder of impulse control and reward dysregulation, but also as a condition rooted in the disruption of core cognitive processes (Hyman, 2005; Yücel et al., 2007; Verdejo-Garcia et al., 2019; Crivelli and Balconi, 2021)—including learning and memory. These dysfunctions involve the pathological over-encoding of drug- or behaviour-related cues, maladaptive reinforcement mechanisms, and reduced responsiveness to natural rewards (Torregrossa et al., 2011; Ngetich et al., 2024). Addiction thereby reflects an aberrant form of learning, in which environmental contingencies are misrepresented and memory systems are recruited to reinforce maladaptive behavioural repertoires.

From a cognitive neuroscience perspective, addiction-related alterations span both declarative and non-declarative memory systems. Declarative memory—especially episodic memory—plays a key role in autobiographical experience and decision-making, while non-declarative memory, such as procedural learning and conditioned responses, governs automatic behaviours and reinforcement learning (Dennis and Perrotti, 2015; Goodman and Packard, 2016). Both systems are known to be compromised by chronic exposure to addictive substances or compulsive behavioural patterns, as confirmed by neuroimaging and neuropsychological evidence (Hyman et al., 2006; Kutlu and Gould, 2016; Barman, 2023; Ngetich et al., 2024). The hippocampus, prefrontal cortex, and striatal circuits—regions central to encoding, consolidation, and retrieval—undergo structural and functional reorganization in the context of repeated drug use (Robbins et al., 2008; Kutlu and Gould, 2016; Goldfarb and Sinha, 2018). Moreover, impairments in synaptic plasticity and long-term potentiation, fundamental to learning mechanisms, have been documented in both substance-related and behavioural addictions (e.g., Bellone and Mameli, 2012; Alotaibi et al., 2024).

Relevant to the present discussion, substance-specific patterns of cognitive impairment have been observed. For instance, chronic stimulant use (e.g., cocaine, amphetamines) has been consistently associated with executive dysfunctions, particularly affecting working memory, attention, and decision-making (Ersche et al., 2006; Ersche and Sahakian, 2007; Verdejo-Garcia and Rubenis, 2020). Alcohol use disorder, in contrast, is often characterized by broad impairments in both declarative and non-declarative memory systems, as well as executive functions such as inhibitory control and planning (Bernardin et al., 2014; Petit et al., 2014; Sachdeva et al., 2016). THC (cannabis) use has been linked with disruption in hippocampal-dependent memory encoding and retrieval, especially among early-onset users (Lundqvist, 2005; Solowij et al., 2011; Meier et al., 2012; Di Ciano et al., 2025). MDMA users often show long-lasting verbal memory deficits due to serotonergic neurotoxicity in medial temporal structures (Gouzoulis-Mayfrank and Daumann, 2009; Wagner et al., 2013).

Critically, these neurocognitive deficits are not limited to substance use. Behavioural addictions—such as gambling disorder, internet addiction, and gaming disorder—also involve disruptions in memory and learning functions, with similar neural substrates affected (Volkow et al., 2010; Zhou et al., 2014; Yau and Potenza, 2015; Weinstein, 2017). Impairments in episodic memory, working memory, and cognitive flexibility are frequently reported, along with impulsive decision-making and maladaptive learning strategies (Potenza, 2014; Antons et al., 2020; Christensen et al., 2023). Gambling disorder, in particular, demonstrates cognitive biases in reward expectation and distorted learning from outcome feedback, which are associated with prefrontal and striatal dysfunctions (Brevers et al., 2013; Wyckmans et al., 2019; Asaoka et al., 2020; García-Castro et al., 2023).

Despite the above-noted theoretical and empirical groundwork, robust and consistent evidence for specific memory deficits—particularly in verbal memory—across addiction types remains limited. As noted in the meta-analysis by Abramovitch et al. (2021), while cognitive dysfunction appears to be a transdiagnostic feature across psychiatric disorders, the specificity and clinical relevance of these deficits vary considerably. Verbal memory impairments, though documented in many studies, are inconsistently observed across clinical cohorts and rarely analysed in detail. Furthermore, methodological heterogeneity—e.g., variations in sample composition, test selection, and analytic focus—limits the generalizability of findings.

In this context, a critical limitation of much of the existing literature is the predominant focus on total performance scores, which often fail to capture the qualitative dimensions of recall behaviour. This issue is partly rooted in the use of traditional cognitive tests in psychiatric settings which, when administered in a superficial or purely quantitative manner, typically yield only global performance indices (e.g., total correct responses), without accounting, as an example, for the type and pattern of errors made by the examinee. Such tools provide a coarse-grained view of cognitive functioning, limiting the ability to differentiate between underlying cognitive processes that may be selectively impaired. By contrast, more recent and structured neurocognitive assessment tools—such as the Battery for Executive Functions in Addiction (BFE-A; Balconi et al., 2022a)—have been specifically developed to offer a more detailed evaluation of cognitive performance. These tools systematically track multiple aspects of task execution, including temporal dynamics and error types, allowing for the computation of process-oriented performance indices.

This approach may help detecting of subtle yet clinically meaningful dysfunctions that would otherwise remain undetected using standard test batteries. Specific types of memory errors—such as intrusions, repetitions, or perseverative responses—might, indeed, reflect discrete cognitive impairments in inhibition, retrieval monitoring, or attentional filtering, and can provide diagnostic information that goes beyond raw performance levels. In keeping with a proper neuropsychological assessment approach, error typologies in neurocognitive testing may reveal disorder-specific patterns of dysfunction, improving clinical differentiation and tailoring of cognitive rehabilitation strategies.

Building on these insights, the present study aims to investigate verbal memory performance and error patterns across several addiction profiles—specifically, stimulant use disorder, alcohol use disorder, cannabis use disorder, and gambling disorder—relative to healthy controls. We used a digitalized neurocognitive screening battery to evaluate immediate and delayed recall performance, with a focus not only on overall memory accuracy but also on specific error types (e.g., intrusion and repetition errors). Our goal is to examine whether distinct addictions are associated with characteristic profiles of memory dysfunction, and whether analysing error typologies can enhance the specificity and clinical value of verbal memory assessments in neuropsychiatric contexts.

Based on the reviewed literature, we hypothesized that individuals showing substance-use disorder as well as gambling disorder would exhibit reduced performance in both immediate and delayed verbal recall compared to healthy controls. In addition to this primary hypothesis, we also expected to observe distinct patterns of recall errors in different addiction profiles, among which more frequent intrusion errors in individuals with alcohol use disorder, reflecting impaired inhibition and interference suppression. Yet, given the limited and inconsistent literature on qualitative aspects of memory in addiction, any hypothesis on error typologies were considered exploratory in nature, aimed at identifying potential disorder-specific cognitive signatures that extend beyond global performance measures.

## Methods

### Participants

The sample consisted of 515 individuals, divided into clinical and healthy subject (HS) cohorts. The HS cohort included 68 participants. The clinical cohorts comprised patients with a diagnosis of Substance Use Disorder (SUD) related to stimulants (Stim; e.g., cocaine, amphetamines, methamphetamine, MDMA), alcohol (Alc), cannabis/tetrahydrocannabinol (THC), or of Gambling Disorder (GD). Table 1 presents the sample size and sociodemographic characteristics of each cohort.

**Table 1:** Size and socio-demographic characteristics of the cohorts and of the total sample.

**Group**	**Group size**	**Sex—M/F (%)**	**Age—Mean (SD); Range**
Stim	240	217 (90%)/23 (10%)	40.5 (9.43); 18–68
Alc	48	23 (48%)/25 (52%)	45.2 (10.96); 18–60
THC	74	61 (82%)/13 (18%)	30.5 (9.93); 18–58
GD	85	71 (83%)/14 *(17%)*	42.6 (14.56); 18–77
HS	68	23 (34%)/45 *(66%)*	37.9 (13.50); 20–60
Total sample	515	395 (77%)/120 *(23%)*	39.5 (11.91); 18–77

Stim, stimulants SUD cohort; Alc, alcohol SUD cohort; THC, cannabis/tetrahydrocannabinol SUD cohort; GD, gambling disorder cohort; HS, healthy subjects cohort.

Inclusion criteria for the HS cohort were: age greater than 18 years; normal or corrected-to-normal vision and hearing. Exclusion criteria for this cohort included: a positive history of neurological or psychiatric disorders; recent or concurrent diagnosis of cerebrovascular events, moderate to severe traumatic brain injury, infectious or inflammatory diseases, neoplastic conditions, or neurodegenerative disorders; clinical instability within 48 h prior to the assessment session; non-compliance or other transient conditions that could compromise data quality; a diagnosis of SUD or GD based on the criteria outlined in the Diagnostic and Statistical Manual of Mental Disorders, 5th edition (DSM-5; APA, 2013); history of recreational psychoactive substance use (excluding alcohol); and first-degree relationships or professional/volunteer experience with individuals diagnosed with a substance-related disorder.

Inclusion criteria for the clinical cohorts were: age greater than 18 years; normal or corrected-to-normal vision and hearing; a diagnosis of SUD or GD according to DSM-5 criteria; and ongoing contact with addiction treatment centers involved in participant recruitment. Exclusion criteria for this cohort included: a positive history of neurological conditions; recent or concurrent diagnosis of cerebrovascular events, moderate to severe traumatic brain injury, infectious or inflammatory diseases, neoplastic conditions, or neurodegenerative disorders; clinical instability within 48 h prior to assessment; non-compliance or other transient conditions that could compromise data quality; and use of either the primary substance of abuse or any other psychoactive substances, or the enaction of the addictive behaviour, within the 72 h preceding assessment.

To ensure ecological validity and representativeness of the clinical cohorts, we adopted an observational case-control design using stratified convenience sampling and snowball sampling methods. These approaches considered clinical history and the aforementioned inclusion/exclusion criteria. Although full matching between clinical and HS groups was only partially achieved by the end of recruitment, this open recruitment strategy allowed for the collection of an ecologically valid sample that reflects the typical patient population accessing addiction services. To minimize potential bias in data interpretation, age and gender were included as covariates in the statistical analyses. Preliminary checks concerning education levels across participants, instead, highlighted no significant difference for means or data variability between groups.

All participants were informed about the purpose of data collection, experimental procedures, and the methods of data processing and storage. Written informed consent was obtained from all participants. The study protocol and procedures were reviewed and approved by the relevant Ethics Committee and were conducted in accordance with the Declaration of Helsinki and its subsequent amendments. Furthermore, data collection, processing, and storage adhered to applicable national and European regulations on privacy and data protection.

### Assessment procedure and materials

Participants were recruited through the primary involvement of the International research center for Cognitive Applied Neuroscience (IrcCAN) at Università Cattolica del Sacro Cuore and the Canzio Addiction Care Service of ASST Fatebenefratelli-Sacco in Milan, with additional support from the Alcoholic and Double Diagnosis Community in Castelfranco Veneto.

A standardized neurocognitive assessment protocol was administered by a team of licensed psychologists trained in psychodiagnostics and neuropsychological testing. The full assessment was conducted in quiet, perceptually neutral examination rooms and completed in a single session lasting approximately 45 min. Test administration was preceded by a structured interview, during which sociodemographic data were collected, along with information regarding any recreational use of alcohol or other substances. When necessary, anamnesis and clinical information were also gathered, including details of substance use history and habits (e.g., primary substance of abuse and any secondary substances).

Participants then completed the Battery for Executive Functions in Addiction—BFE-A (Balconi et al., 2022a), a digitized neurocognitive screening battery composed of seven subtests. These subtests evaluate: verbal learning and short-/long-term memory, working memory, verbal and non-verbal cognitive flexibility, focused attention, attention regulation and interference suppression, and inhibitory control. Scoring of participants' performance on each subtest was conducted by expert examiners and subsequently double-checked by a second expert in neuropsychological assessment, acting as an independent, blinded reviewer.

In line with our primary research aim and the relevant literature reviewed, the present brief research report focuses on the outcomes of the subtests targeting verbal learning and short-/long-term memory. Specifically, we examine results from the Verbal Memory Test (VMT) of the BFE-A, which assesses verbal learning and memory processes through immediate and delayed serial recall tasks. The VMT is designed to evaluate encoding, consolidation, and retrieval of verbally presented auditory stimuli. During administration, participants are presented with a list of 15 words, repeated 5 times. After each presentation, they are asked to recall the words verbally trying to respect the original order of words presentation. A delayed serial recall is then requested 10 min later, without any cues.

Performance and error metrics are recorded for both immediate and delayed recall phases and include: number of correctly recalled words (Correct Responses Index, CR_i_), number of words recalled in the correct serial position (Position Index, P_i_), intrusion errors (Err_Int_), and repetition errors (Err_Rip_). Additional technical details regarding the nature and computation of those scores can be found in the official BFE-A manual (Balconi et al., 2022a). Additional information on the subtest structure could be found in the official BFE-A manual and in the following reports (Balconi and Crivelli, 2021; Balconi et al., 2022b; Crivelli et al., 2024).

### Data analyses

Performance and error metrics—namely, number of correctly recalled words, number of words recalled in the correct serial position, intrusion errors, and repetition errors—for both immediate recall and delayed recall trials were analysed through ANCOVA models including Group (HS, Stim, Alc, THC, GD) as between-subject independent factor, as well as controlling for sex and age as covariates. Partial eta squared ($\eta_{p}^{2}$) was used as the effect size estimator for the main effect of group, representing the proportion of variance in performance uniquely explained by cohort membership, after accounting for covariates. *Post-hoc* pairwise comparisons were conducted on estimated marginal means (EMMs), adjusted for age and sex. Tukey's correction was applied to control for multiple comparisons and maintain the family-wise error rate. For each pairwise comparison, Cohen's d was also calculated to estimate effect size, providing a standardized measure of the magnitude of group differences. Effect size estimates were deemed as small when 0.01 ≤ $\eta_{p}^{2}$ < 0.06 or |0.20| ≤ d < |0.50|, medium when 0.06 ≤ $\eta_{p}^{2}$ < 0.14 or |0.50| ≤ d < |0.80|, and large when $\eta_{p}^{2}$ ≥ 0.14 or d ≥ |0.80|, in agreement with Cohen's norms (Cohen, 1988). Threshold for statistical significance was set to α = 0.05.

Also, in order to increase the accuracy of statistical inference and to check whether the actual sample size could be deemed as sufficient to test study hypotheses, an *a priori* power analysis was conducted using G^*^Power (version 3.1.9.7). Results indicated the required sample size to achieve 80% power for detecting a medium effect (*f* = 0.25), at a significance criterion of α = 0.05, was *N* = 196 for an ANCOVA model with one independent between-subjects factor (Group, 5 levels) and two covariates. Accounting for a potential attrition rate of 20%, the minimum required sample size would be *N* = 235. Thus, the considered the obtained sample size (*N* = 515) adequate to test the study hypotheses.

## Results

**Figure 1:**
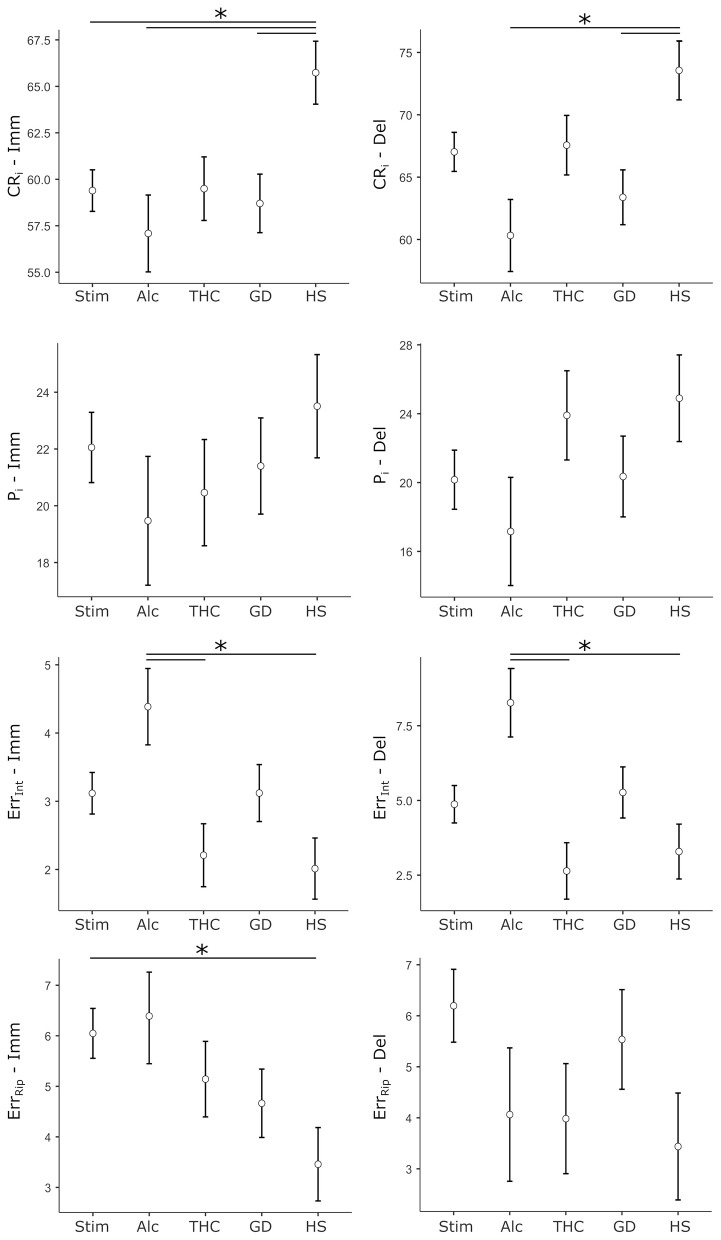
Cohorts' data for performance metrics of the Verbal Memory Test. Dots represent the cohort mean score, whiskers represent ± 1 SE. Stars mark statistically significant pairwise comparisons. Left column: scores relative to immediate recall trials (Imm). Right column: scores relative to delayed recall trials (Del). CR_i_, correct responses index; P_i_, position index; Err_Int_, intrusion errors; Err_Rip_, repetition errors; Stim, stimulants SUD cohort; Alc, alcohol SUD cohort; THC, cannabis/tetrahydrocannabinol SUD cohort; GD, gambling disorder cohort; HS - healthy subjects cohort.

To examine group differences in cognitive performance across immediate and delayed recall conditions, ANCOVAs models were run with Group (HS, Stim, Alc, THC, and GD) as the between-subject factor, and age and sex entered as covariates. Dependent variables were four performance indices—CR_i_, P_i_, Err_Int_, and Err_Rip_ scores—measured during immediate (Imm) and delayed (Del) recall trials. Figure 1 reports a graphical depiction of cohorts' data for each performance metrics.

### Immediate recall

For CR_i_-Imm, the analysis revealed a significant main effect of Group (*F*[4, 489] = 3.36, *p* = 0.010, $\eta_{\text{p}}^{2}$ = 0.027). Age was a significant covariate (*F*[1, 489] = 26.73, *p* < .001, $\eta_{\text{p}}^{2}$ = 0.052), while sex did not contribute significantly (*F*[1, 489] = 0.69, *p* = 0.406). *Post-hoc* comparisons showed that, compared to the HS group, the Stim group (*p* = 0.024, d = 0.47), the Alc group (*p* = 0.012, d = 0.64), and the GD group (*p* = 0.028, d = 0.52) had significantly lower scores. No other pairwise comparisons reached significance.

The ANCOVA for P_i_-Imm did not show a significant main effect of Group (*F*[4, 466] = 0.66, *p* = 0.617). Age was again found to be a significant predictor of performance (*F*[1, 466] = 43.10, *p* < 0.001, $\eta_{\text{p}}^{2}$ = 0.085), while sex was not (*F*[1, 466] = 0.44, *p* = 0.506).

In contrast, the analysis for Err_Int_-Imm showed a significant main effect of Group (*F*[4, 466] = 3.43, *p* = 0.009, $\eta_{\text{p}}^{2}$ = 0.029). Neither age (*F*[1, 466] = 0.13, *p* = 0.719) nor sex (*F*[1, 466] = 0.58, *p* = 0.445) were significant predictors. *Post-hoc* comparisons indicated that the Alc group performed significantly worse than both the THC group (*p* = 0.027, d = 0.61) and the HS group (*p* = 0.009, d = 0.67), suggesting selective deficits in inhibitory recall.

The Err_Rip_-Imm model also showed a significant Group effect (*F*[4, 466] = 2.86, *p* = 0.023, $\eta_{\text{p}}^{2}$ = 0.024). Age emerged as a significant covariate (*F*[1, 466] = 5.41, *p* = 0.020, $\eta_{\text{p}}^{2}$ = 0.011), while sex was not (*F*[1, 466] = 1.24, *p* = 0.267). *Post-hoc* analysis revealed that the Stim group had significantly higher Err_Rip_-Imm scores than the HS group (*p* = 0.040, d = 0.45), reflecting selectively altered performance in such clinical cohort.

### Delayed recall

Turning to the delayed recall trial, the ANCOVA for CR_i_-Del showed a robust main effect of Group (*F*[4, 487] = 3.86, *p* = 0.004, $\eta_{\text{p}}^{2}$ = 0.031). Age and sex were also significant predictors (age: *F*[1, 487] = 58.84, *p* < 0.001, $\eta_{\text{p}}^{2}$ = 0.108; sex: *F*[1, 487] = 5.41, *p* = 0.020, $\eta_{\text{p}}^{2}$ = 0.011). *Post-hoc* comparisons indicated that the Alc group (*p* = 0.004, d = 0.71) and the GD group (*p* = 0.020, d = 0.54), specifically, scored significantly lower than the HS group.

The P_i_-Del model did not reveal a significant main effect of Group (*F*[4, 464] = 1.32, *p* = 0.263) nor of sex (*F*[1, 464] = 0.75, *p* = 0.387), but an age effect (*F*[1, 464] = 34.33, *p* < 0.001, $\eta_{\text{p}}^{2}$ = 0.069).

For Err_Int_-Del, Group differences were statistically significant (*F*[4, 464] = 4.27, *p* = 0.002, $\eta_{\text{p}}^{2}$ = 0.036). Neither age (*F*[1, 464] = 2.11, *p* = 0.147) nor sex (*F*[1, 464] = 2.31, *p* = 0.129) were significant. *Post-hoc* tests revealed that the Alcohol group performed worse than the THC group (*p* = 0.002, d = 0.77) and the HS group (*p* = 0.007, d = 0.68).

Finally, the ANCOVA for Err_Rip_-Del showed no significant main effect of Group (*F*(4, 464) = 1.68, *p* = 0.154). Neither sex (*F*[1, 464] = 1.95, *p* = 0.164) nor age (*F*[1, 464] = 0.40, *p* = 0.529) significantly predicted performance in this model.

## Discussion

The present study aimed at exploring verbal memory impairments across individuals with various SUDs—including stimulant, alcohol, and cannabis use disorders—and GD, compared to healthy controls, using a digitalized neurocognitive screening tool. In doing so, we aimed not only to examine global performance in immediate and delayed verbal recall, but also to assess the diagnostic relevance of specific recall error types, which remain a relatively underexplored yet potentially informative dimension in addiction-related cognitive assessment, especially when comparing substance-related and behavioural addictions.

Our findings reveal a multifaceted pattern of impairment that is broadly consistent with the hypothesis of domain-specific disruptions in memory processes across addiction profiles. These impairments primarily affected the accuracy of both immediate and delayed verbal recall across all the addiction disorders, while also uncovering distinct profiles in intrusion and repetition errors—thereby offering a more graded perspective on their cognitive consequences and correlates.

In the immediate recall condition, correctly recalled item scores were significantly lower among participants with stimulant use disorder, alcohol use disorder, and GD, relative to healthy controls. These medium-effect size deficits suggest that early-stage verbal encoding processes—particularly those relying on focused attention and controlled learning—are consistently compromised in these clinical cohorts. The cognitive load associated with rapid verbal input and the requirement for strategic encoding may overwhelm the executive capacities of individuals with addiction, aligning with models of impaired cognitive control and prefrontal dysfunction (Ersche et al., 2006; Goldstein and Volkow, 2011; Zhou et al., 2014; Goodman and Packard, 2016; Uhl et al., 2019).

Interestingly, performance on the position index, a measure of recall organization based on serial order, did not differ significantly across groups but was uniquely affected by participants' age. This dissociation suggests that while the amount of recalled information is reduced in clinical cohorts, the structural organization of what is recalled might remain relatively preserved when tapping on short-term memory and immediate retrieval stage. Such a pattern supports the notion that executive and attentional resources may be selectively taxed during encoding, without necessarily disrupting the mnemonic schema or strategy used to organize information. Nonetheless, it is also worth noting that additional insight on the adaptive (or maladaptive) mechanisms and strategies used to solve the memory task could come from a deeper exploration of the various strategies—besides sequential recall—used by the examinees during memorization, and that it might help to better interpret the observed lack of differences for performance on the position index across groups. By taking into account such further data, it would be possible to complement the evaluation by identifying participant's naturally preferred strategy for coping with recalling of words and by assessing, for example, their ability to inhibit such dominant strategy and promote a specific requested one.

Error analysis, then, provided more specific insight into the mechanisms of memory-related dysfunction in addiction. Intrusion errors were particularly elevated in the patients presenting alcohol use disorders, suggesting compromised inhibitory control and a failure to suppress irrelevant or competing memory traces—likely reflective of prefrontal disinhibition and poor monitoring processes (De Beni et al., 1998; De Beni and Palladino, 2004; Fischer-Baum and McCloskey, 2015). In contrast, repetition errors were significantly more frequent in the patients presenting stimulant use disorders, which may indicate impulsive recall tendencies and insufficient metacognitive control on the immediate retrieval process from short-term memory. This finding aligns with the characteristic neurocognitive profile of stimulant use, which often includes heightened impulsivity and reduced feedback sensitivity (Fillmore et al., 2005; Jovanovski et al., 2005; Gouzoulis-Mayfrank and Daumann, 2009; Balconi et al., 2014; Almeida et al., 2017).

Crucially, these distinct error types may correspond to disruptions in specific subcomponents of memory processing. While intrusions likely reflect deficits in attentional filtering, interference suppression, or inhibitory control, repetitions are more indicative of a breakdown in internal monitoring and memory updating—functions typically attributed to dorsolateral prefrontal networks and executive working memory (Koriat et al., 1988; Koriat and Goldsmith, 1996; Benjamin, 2008; Castel et al., 2016). As such, error typologies may provide an important, often overlooked lens for differentiating the cognitive signatures of various addiction disorders.

Notably, in the delayed recall trial—which primarily taxes long-term memory—impairments in verbal memory persisted and, in some cases, became more pronounced. Participants in the alcohol and GD groups showed significantly lower scores compared to healthy participants on the delayed correctly recalled items index, suggesting that these populations experience marked difficulties not only in encoding but also in memory consolidation and retrieval. This is consistent with prior findings linking alcohol use to long-term hippocampal and frontal damage, and with the limited evidence of altered information processing and memory trace reconsolidation in GD (Volkow et al., 2010; Potenza, 2014; Zhou et al., 2014; Asaoka et al., 2020).

Again, the position index did not differ significantly between groups, reinforcing the notion that sequential recall strategies may remain intact despite global memory impairments. However, patients presenting alcohol-related addiction continued to show elevated intrusion errors during delayed recall, a pattern that suggests persistent interference from irrelevant memory content and deficient suppression mechanisms—possibly reflecting enduring disruption in prefrontal inhibitory circuits. In contrast, repetition errors in the delayed recall trial did not differ significantly across groups, indicating that perseverative recall tendencies may be more transient or context-dependent, and less reliably engaged during longer retention intervals.

As a final note, we deem worth noting that the performance of individuals with GD largely mirrored that of substance-using groups, particularly in terms of reduced immediate and delayed recall accuracy. This supports the view that behavioural and substance-related addictions share overlapping neurocognitive dysfunctions, including impairments in reward-based learning, episodic memory, and executive control (Potenza, 2008; Goldstein and Volkow, 2011; Brand et al., 2019; Uhl et al., 2019; Ngetich et al., 2024). However, the absence of elevated error rates in GD suggests a partially distinct cognitive profile. Unlike patients showing alcohol-related and stimulant-related addiction—where deficits were characterized not only by poor recall accuracy but also by heightened and specific errors —individuals with GD showed impairment mainly in terms of recall quantity, not quality. This may reflect more subtle disruptions in encoding or consolidation, without the accompanying disinhibition or monitoring deficits observed in SUD groups. Such a distinction is important, as it underscores that while GD and SUDs may converge in terms of overall performance decline, the cognitive mechanisms underlying those impairments might differ. This aligns with neurobiological models proposing that while both types of addiction involve dysfunctional reinforcement learning and prefrontal-striatal disconnection, the extent and nature of memory-related dysfunctions may be modulated by the presence or absence of neurotoxic substance exposure (Potenza, 2008; Gouzoulis-Mayfrank and Daumann, 2009; Yau and Potenza, 2015; Weinstein, 2017; Brand et al., 2019; Uhl et al., 2019).

## Study limitations

While the present study offers valuable insights into verbal memory performance and error profiles across various substance use and behavioural addiction cohorts, several limitations should be acknowledged. First, the cross-sectional design limits our ability to infer causal relationships between addictive behaviours and cognitive dysfunctions. Longitudinal studies are needed to clarify the temporal dynamics of memory impairment, particularly regarding the persistence or reversibility of deficits following abstinence or therapeutic intervention. Second, although our sample was ecologically valid and heterogeneous, group sizes across clinical cohorts were unequal, and the use of convenience and snowball sampling may introduce selection bias. Additionally, while age and sex were statistically controlled, other potential confounding factors—such as duration and severity of addiction, polysubstance use, psychiatric comorbidities, or medication status—were not comprehensively accounted for, potentially affecting the observed neurocognitive profiles. Third, the focus on a single memory task, albeit part of a broader neurocognitive battery, limits the generalizability of findings to other types of memory (e.g., visuospatial, prospective) or learning domains. Future studies employing a broader range of tasks could better delineate the domain-specificity of memory dysfunctions in different addiction types.

## Conclusions

To conclude, our findings, taken together, highlight the potential for improving assessment outcomes of combining performance-based measures with detailed error analysis in verbal memory tasks, to better characterize the cognitive profiles of individuals with addiction. Rather than relying solely on global recall scores, parsing out the types of errors—intrusions vs. repetitions—offers a more graded view of the underlying cognitive deficits and may aid in differential diagnosis, intervention planning, and treatment monitoring. This approach is consistent with recent calls for a more functionally informed, domain-specific neuropsychological assessment framework in addiction research (Abramovitch et al., 2021; Crivelli and Balconi, 2021). The pattern of findings also supports the view that different addiction phenotypes—while sharing commonalities in memory dysfunction—may be differentiated by the specific cognitive processes they affect. Alcohol-related disorders appear to produce diffuse deficits across encoding, inhibition, and retrieval; stimulant use is marked by heightened perseveration and impulsive recall; and GD exhibits more restricted impairments, possibly reflecting a different balance between habitual and goal-directed control in memory expression. These distinctions carry potential relevance for tailored cognitive rehabilitation protocols. For instance, interventions focusing on error awareness and inhibitory control might be more beneficial in alcohol or stimulant users, whereass strategies targeting memory consolidation and goal-directed recall may be prioritized for individuals with GD.

## Data Availability

The datasets presented in this article are not readily available due to the sensitive nature of data. Reasonable requests to access the datasets should be directed to Davide Crivelli, davide.crivelli@unciatt.it.

## References

[B1] Abramovitch A. Short T. Schweiger A. (2021). The c factor: cognitive dysfunction as a transdiagnostic dimension in psychopathology. Clin. Psychol. Rev. 86:102007. doi: 10.1016/j.cpr.2021.102007 33864968

[B2] Almeida P. P. de Araujo Filho G. M. Malta S. M. Laranjeira R. R. Marques A. C. R. P. Bressan R. A. . (2017). Attention and memory deficits in crack-cocaine users persist over four weeks of abstinence. J. Subst. Abuse Treat 81, 73–78. doi: 10.1016/j.jsat.2017.08.002 28847458

[B3] Alotaibi S. Emara A. M. Elsisi H. A. (2024). Mechanisms of psychiatric disorders induced by Amphetamines: a comprehensive review. Int. J. Sci. Res. Arch. 11, 260–274. doi: 10.30574/ijsra.2024.11.1.0007

[B4] Antons S. Brand M. Potenza M. N. (2020). Neurobiology of cue-reactivity, craving, and inhibitory control in non-substance addictive behaviors. J. Neurol. Sci. 415:116952. doi: 10.1016/j.jns.2020.116952 32534370

[B5] APA (2013). Diagnostic and Statistical Manual of Mental Disorders. Fifth Edition. Arlington. VA: American Psychiatric Association.

[B6] Asaoka Y. Won M. Morita T. Ishikawa E. Goto Y. (2020). Higher risk taking and impaired probability judgment in behavioral addiction. Int. J. Neuropsychopharmacol. 23, 662–672. doi: 10.1093/ijnp/pyaa044 32574348 PMC7727479

[B7] Balconi M. Crivelli D. (2021). “The assessment of executive functions: a new neuropsychological tool for addiction,” in Advances in Substance and Behavioral Addiction. The Role of Executive Functions, eds. Balconi M. Campanella S. (Cham: Springer), 61–85. doi: 10.1007/978-3-030-82408-2_3

[B8] Balconi M. Finocchiaro R. Campanella S. (2014). Reward sensitivity, decisional bias, and metacognitive deficits in cocaine drug addiction. J. Addict. Med. 8, 399–406. doi: 10.1097/ADM.0000000000000065 25303980

[B9] Balconi M. Losasso D. Balena A. Crivelli D. (2022a). BFE-A - Batteria per le Funzioni Esecutive nell'Addiction (Battery for Exective Functions in Addiction). Firenze: Giunti Psychometrics.

[B10] Balconi M. Losasso D. Balena A. Crivelli D. (2022b). Neurocognitive impairment in addiction: a digital tool for executive function assessment. Front. Psychiatry 13:955277. doi: 10.3389/fpsyt.2022.955277 36276307 PMC9579426

[B11] Barman K. P. (2023). A brief review of the relationship between addiction and memory systems. World J. Neurosci. 13, 151–159. doi: 10.4236/wjns.2023.133010

[B12] Bellone C. Mameli M. (2012). mGluR-dependent synaptic plasticity in drug-seeking. Front. Pharmacol. 3:159. doi: 10.3389/fphar.2012.00159 22969723 PMC3428011

[B13] Benjamin A. S. (2008). “Memory is more than just remembering: strategic control of encoding, accessing memory, and making decisions,” in Skill and strategy in memory use, eds. Benjamin A. S. Ross B. H. (San Diego, CA, US: Elsevier Academic Press), 175–223. doi: 10.1016/S0079-7421(07)48005-7

[B14] Bernardin F. Maheut-Bosser A. Paille F. (2014). Cognitive impairments in alcohol-dependent subjects. Front Psychiatry 5:78. doi: 10.3389/fpsyt.2014.00078 25076914 PMC4099962

[B15] Brand M. Wegmann E. Stark R. Müller A. Wölfling K. Robbins T. W. . (2019). The Interaction of Person-Affect-Cognition-Execution (I-PACE) model for addictive behaviors: update, generalization to addictive behaviors beyond internet-use disorders, and specification of the process character of addictive behaviors. Neurosci. Biobehav. Rev. 104, 1–10. doi: 10.1016/j.neubiorev.2019.06.032 31247240

[B16] Brevers D. Bechara A. Cleeremans A. Noël X. (2013). Iowa Gambling Task (IGT): twenty years after—gambling disorder and IGT. Front. Psychol 4:665. doi: 10.3389/fpsyg.2013.00665 24137138 PMC3786255

[B17] Castel A. D. Middlebrooks C. D. McGillivray S. (2016). “Monitoring memory in old age: impaired, spared, and aware,” in The Oxford handbook of metamemory., eds. Dunlosky J. Tauber S. K. (New York, NY: Oxford University Press), 519–535. doi: 10.1093/oxfordhb/9780199336746.013.3

[B18] Christensen E. Brydevall M. Albertella L. Samarawickrama S. K. Yücel M. Lee R. S. C. (2023). Neurocognitive predictors of addiction-related outcomes: a systematic review of longitudinal studies. Neurosci. Biobehav. Rev. 152:105295. doi: 10.1016/j.neubiorev.2023.105295 37391111

[B19] Cohen J. (1988). Statistical Power Analysis for the Behavioral Sciences., II. Hillsdale, NJ: Lawrence Erlbaum Associates.

[B20] Crivelli D. Balconi M. (2021). “Psychopathology of EFs,” in Advances in Substance and Behavioral Addiction. The Role of Executive Functions, eds. Balconi M. Campanella S. (Cham: Springer), 41–59. doi: 10.1007/978-3-030-82408-2_2

[B21] Crivelli D. Balena A. Losasso D. Balconi M. (2024). Screening executive functions in substance-use disorder: first evidence from testing of the Battery for Executive Functions in Addiction (BFE-A). Int. J. Ment. Health Addict. 22, 1315–1332. doi: 10.1007/s11469-022-00928-5

[B22] De Beni R. Palladino P. (2004). Decline in working memory updating through ageing: intrusion error analyses. Memory 12, 75–89. doi: 10.1080/09658210244000568 15098622

[B23] De Beni R. Palladino P. Pazzaglia F. Cornoldi C. (1998). Increases in intrusion errors and working memory deficit of poor comprehenders. Q. J. Exp. Psychol. A 51, 305–320. doi: 10.1080/713755761 9621841

[B24] Dennis T. S. Perrotti L. I. (2015). Erasing drug memories through the disruption of memory reconsolidation: a review of glutamatergic mechanisms. J. Appl. Biobehav. Res. 20, 101–129. doi: 10.1111/jabr.12031

[B25] Di Ciano P. Zhao S. Kaduri P. Patel S. Bhakta K. Wickens C. M. . (2025). Effects of naturalistic doses of cannabis edibles on cognition and association with blood THC. Psychopharmacology doi: 10.1007/s00213-025-06863-2 40748375

[B26] Ersche K. D. Clark L. London M. Robbins T. W. Sahakian B. J. (2006). Profile of executive and memory function associated with amphetamine and opiate dependence. Neuropsychopharmacology 31, 1036–1047. doi: 10.1038/sj.npp.1300889 16160707 PMC1867318

[B27] Ersche K. D. Sahakian B. J. (2007). The neuropsychology of amphetamine and opiate dependence: implications for treatment. Neuropsychol. Rev. 17, 317–336. doi: 10.1007/s11065-007-9033-y 17690986 PMC3639428

[B28] Fillmore M. T. Kelly T. H. Martin C. A. (2005). Effects of d-amphetamine in human models of information processing and inhibitory control. Drug Alcohol Depend 77, 151–159. doi: 10.1016/j.drugalcdep.2004.07.013 15664716 PMC3201830

[B29] Fischer-Baum S. McCloskey M. (2015). Representation of item position in immediate serial recall: evidence from intrusion errors. J. Exp. Psychol. Learn Mem. Cogn. 41, 1426–1446. doi: 10.1037/xlm0000102 25730307

[B30] García-Castro J. Cancela A. Cárdaba M. A. M. (2023). Neural cue-reactivity in pathological gambling as evidence for behavioral addiction: a systematic review. Curr. Psychol. 42, 28026–28037. doi: 10.1007/s12144-022-03915-0 36373116 PMC9638381

[B31] Goldfarb E. V. Sinha R. (2018). Drug-induced glucocorticoids and memory for substance use. Trends Neurosci. 41, 853–868. doi: 10.1016/j.tins.2018.08.005 30170822 PMC6204074

[B32] Goldstein R. Z. Volkow N. D. (2011). Dysfunction of the prefrontal cortex in addiction: neuroimaging findings and clinical implications. Nat. Rev. Neurosci. 12, 652–669. doi: 10.1038/nrn3119 22011681 PMC3462342

[B33] Goodman J. Packard M. G. (2016). Memory systems and the addicted brain. Front. Psychiatry 7:24. doi: 10.3389/fpsyt.2016.00024 26941660 PMC4766276

[B34] Gouzoulis-Mayfrank E. Daumann J. (2009). Neurotoxicity of drugs of abuse - the case of methylenedioxy amphetamines (MDMA, ecstasy), and amphetamines. Dialogues Clin. Neurosci. 11, 305–317. doi: 10.31887/DCNS.2009.11.3/egmayfrank 19877498 PMC3181923

[B35] Hyman S. E. (2005). Addiction: a disease of learning and memory. Am. J. Psychiatry 162, 1414–1422. doi: 10.1176/appi.ajp.162.8.1414 16055762

[B36] Hyman S. E. Malenka R. C. Nestler E. J. (2006). Neural mechanisms of addiction: the role of reward-related learning and memory. Annu. Rev. Neurosci. 29, 565–598. doi: 10.1146/annurev.neuro.29.051605.113009 16776597

[B37] Jovanovski D. Erb S. Zakzanis K. K. (2005). Neurocognitive deficits in cocaine users: a quantitative review of the evidence. J. Clin. Exp. Neuropsychol. 27, 189–204. doi: 10.1080/13803390490515694 15903150

[B38] Koriat A. Ben-Zur H. Sheffer D. (1988). Telling the same story twice: output monitoring and age. J. Mem. Lang. 27, 23–39. doi: 10.1016/0749-596X(88)90046-0

[B39] Koriat A. Goldsmith M. (1996). Monitoring and control processes in the strategic regulation of memory accuracy. Psychol. Rev. 103, 490–517. doi: 10.1037/0033-295X.103.3.490 8759045

[B40] Kutlu M. G. Gould T. J. (2016). Effects of drugs of abuse on hippocampal plasticity and hippocampus-dependent learning and memory: contributions to development and maintenance of addiction. Learn. Mem. 23, 515–533. doi: 10.1101/lm.042192.116 27634143 PMC5026208

[B41] Lundqvist T. (2005). Cognitive consequences of cannabis use: comparison with abuse of stimulants and heroin with regard to attention, memory and executive functions. Pharmacol. Biochem. Behav. 81, 319–330. doi: 10.1016/j.pbb.2005.02.017 15925403

[B42] Meier M. H. Caspi A. Ambler A. Harrington H. Houts R. Keefe R. S. E. . (2012). Persistent cannabis users show neuropsychological decline from childhood to midlife. Proc. Natl. Acad. Sci. 109, E2657–E2664. doi: 10.1073/pnas.1206820109 22927402 PMC3479587

[B43] Ngetich R. Villalba-García C. Soborun Y. Vékony T. Czakó A. Demetrovics Z. . (2024). Learning and memory processes in behavioural addiction: a systematic review. Neurosci. Biobehav. Rev. 163:105747. doi: 10.1016/j.neubiorev.2024.105747 38870547

[B44] Petit G. Cimochowska A. Kornreich C. Hanak C. Verbanck P. Campanella S. (2014). Neurophysiological correlates of response inhibition predict relapse in detoxified alcoholic patients: some preliminary evidence from event-related potentials. Neuropsychiatr. Dis. Treat 10, 1025–1037. doi: 10.2147/NDT.S61475 24966675 PMC4062548

[B45] Potenza M. N. (2008). The neurobiology of pathological gambling and drug addiction: an overview and new findings. Philos. Trans. R Soc. Lond. B Biol. Sci. 363, 3181–3189. doi: 10.1098/rstb.2008.0100 18640909 PMC2607329

[B46] Potenza M. N. (2014). The neural bases of cognitive processes in gambling disorder. Trends Cogn. Sci. 18, 429–438. doi: 10.1016/j.tics.2014.03.007 24961632 PMC4112163

[B47] Robbins T. W. Ersche K. D. Everitt B. J. (2008). Drug addiction and the memory systems of the brain. Ann. N Y Acad. Sci. 1141, 1–21. doi: 10.1196/annals.1441.020 18991949

[B48] Sachdeva A. Chandra M. Choudhary M. Dayal P. Anand K. S. (2016). Alcohol-related dementia and neurocognitive impairment: a review study. Int. J. High Risk Behav. Addict. 5:e27976. doi: 10.5812/ijhrba.27976 27818965 PMC5086415

[B49] Solowij N. Jones K. A. Rozman M. E. Davis S. M. Ciarrochi J. Heaven P. C. L. . (2011). Verbal learning and memory in adolescent cannabis users, alcohol users and non-users. Psychopharmacology (Berl) 216, 131–144. doi: 10.1007/s00213-011-2203-x 21328041

[B50] Torregrossa M. M. Corlett P. R. Taylor J. R. (2011). Aberrant learning and memory in addiction. Neurobiol. Learn. Mem. 96, 609–623. doi: 10.1016/j.nlm.2011.02.014 21376820 PMC3138832

[B51] Uhl G. R. Koob G. F. Cable J. (2019). The neurobiology of addiction. Ann. N. Y. Acad. Sci. 1451, 5–28. doi: 10.1111/nyas.13989 30644552 PMC6767400

[B52] Verdejo-Garcia A. Garcia-Fernandez G. Dom G. (2019). Cognition and addiction. Dialog. Clin. Neurosci. 21, 281–290. doi: 10.31887/DCNS.2019.21.3/gdom 31749652 PMC6829168

[B53] Verdejo-Garcia A. Rubenis A. J. (2020). “Cognitive deficits in people with stimulant use disorders,” in Cognition and Addiction. A Researcher's Guide from Mechanisms Towards Interventions, ed. Verdejo-Garcia A. (London: Academic Press), 155–163. doi: 10.1016/B978-0-12-815298-0.00011-3

[B54] Volkow N. D. Wang G. Fowler J. S. Tomasi D. Telang F. Baler R. (2010). Addiction: decreased reward sensitivity and increased expectation sensitivity conspire to overwhelm the brain's control circuit. BioEssays 32, 748–755. doi: 10.1002/bies.201000042 20730946 PMC2948245

[B55] Wagner D. Becker B. Koester P. Gouzoulis-Mayfrank E. Daumann J. (2013). A prospective study of learning, memory, and executive function in new MDMA users. Addiction 108, 136–145. doi: 10.1111/j.1360-0443.2012.03977.x 22831704

[B56] Weinstein A. M. (2017). An update overview on brain imaging studies of internet gaming disorder. Front. Psychiatry 8:185. doi: 10.3389/fpsyt.2017.00185 29033857 PMC5626837

[B57] Wyckmans F. Otto A. R. Sebold M. Daw N. Bechara A. Saeremans M. . (2019). Reduced model-based decision-making in gambling disorder. Sci. Rep. 9:19625. doi: 10.1038/s41598-019-56161-z 31873133 PMC6927960

[B58] Yau Y. H. C. Potenza M. N. (2015). Gambling disorder and other behavioral addictions. Harv. Rev. Psychiatry 23, 134–146. doi: 10.1097/HRP.0000000000000051 25747926 PMC4458066

[B59] Yücel M. Lubman D. I. Solowij N. Brewer W. J. (2007). Understanding drug addiction: a neuropsychological perspective. Aust. N. Z. J. Psychiatry 41, 957–968. doi: 10.1080/00048670701689444 17999268

[B60] Zhou Z. Zhu H. Li C. Wang J. (2014). Internet addictive individuals share impulsivity and executive dysfunction with alcohol-dependent patients. Front. Behav. Neurosci. 8:288. doi: 10.3389/fnbeh.2014.00288 25202248 PMC4142341

